# A Diet with Amikacin Changes the Bacteriobiome and the Physiological State of *Galleria mellonella* and Causes Its Resistance to *Bacillus thuringiensis*

**DOI:** 10.3390/insects14110889

**Published:** 2023-11-17

**Authors:** Olga V. Polenogova, Tatyana N. Klementeva, Marsel R. Kabilov, Tatyana Y. Alikina, Anton V. Krivopalov, Natalya A. Kruykova, Viktor V. Glupov

**Affiliations:** 1 Institute of Systematics and Ecology of Animals, Siberian Branch of Russian Academy of Sciences, Novosibirsk 630091, Russia; red.klen@yandex.ru (T.N.K.); krivopalov@gmail.com (A.V.K.); dragonfly6@yandex.ru (N.A.K.); skif61@list.ru (V.V.G.); 2 Institute of Chemical Biology and Fundamental Medicine, Siberian Branch of Russian Academy of Sciences, Novosibirsk 630090, Russia; kabilov@niboch.nsc.ru (M.R.K.); alikina@niboch.nsc.ru (T.Y.A.)

**Keywords:** wax moth, antibiotic, microbiota, amikacin

## Abstract

**Simple Summary:**

The insect gut microbiota plays a crucial role in the host’s resistance to pathogenic microorganisms and toxins. Resident microorganisms may persist for a long time and maintain a certain location due to the synthesis of antimicrobial agents (including antibiotics). We consider it one of the adaptation mechanisms of microorganisms in various communities. Not only do the uncontrolled use of antibiotics and changes in environmental conditions drastically alter the microbiota structure and give rise to resistant microorganisms, but they also cause a number of alterations in the host’s physiology and its sensitivity to pathogens. This study opens up new prospects for further research into antibiotic-resistant symbiotic microorganisms, their benefits for persistence in the dynamic environment of the insect’s gut, and the conditions necessary for changing their strategy and manifestation of virulent properties.

**Abstract:**

Environmental pollution with antibiotics can cause antibiotic resistance in microorganisms, including the intestinal microbiota of various insects. The effects of low-dose aminoglycoside antibiotic (amikacin) on the resident gut microbiota of *Galleria mellonella*, its digestion, its physiological parameters, and the resistance of this species to bacteria *Bacillus thuringiensis* were investigated. Here, 16S rDNA analysis revealed that the number of non-dominant *Enterococcus mundtii* bacteria in the eighteenth generation of the wax moth treated with amikacin was increased 73 fold compared to *E. faecalis*, the dominant bacteria in the native line of the wax moth. These changes were accompanied by increased activity of acidic protease and glutathione-*S*-transferase in the midgut tissues of larvae. Ultra-thin section electron microscopy detected no changes in the structure of the midgut tissues. In addition, reduced pupa weight and resistance of larvae to *B. thuringiensis* were observed in the eighteenth generation of the wax moth reared on a diet with amikacin. We suggest that long-term cultivation of wax moth larvae on an artificial diet with an antibiotic leads to its adaptation due to changes in both the gut microbiota community and the physiological state of the insect organism.

## 1. Introduction

Environmental pollution with antibiotics quite predictably leads to the emergence of antibiotic-resistant microorganisms, including various insect microbiota. Antibiotic resistance developed by bacteria affects the dynamics of insect populations and their adaptive capabilities both to other xenobiotics and environmental factors. Unfortunately, there are virtually no studies addressing this issue; however, the use of insects as test objects for pharmacological and toxicological studies could be a promising direction for research [1]. Meanwhile, antibiotics are widely used for investigating insect microbiota or their role for some specific bacteria in the host microbiome [2,3,4,5]. Even small amounts of substances exhibiting antibiotic activity may cause the spread of resistance genes and the emergence of multidrug-resistance in microorganisms. The development of resistant forms of microbiota in the insect gut can cause changes in the metabolic rate of the host species [6,7]. Finally, an imbalance in the gut microbiota may alter the activity of digestive enzymes and food digestibility, as well as reduce host viability and breeding [8,9,10]. Toxicosis progression is often accompanied by an increased count of bacteria belonging to certain groups and an imbalance in the microbiota [11,12,13,14]. The presence of certain microbiota in the gut definitely facilitates the host’s adaptation to different diets and metabolites [15,16,17] and plays a significant role in toxin degradation [18,19,20].

*Bacillus thuringiensis* (*Bt*) is a prevalent soil bacterium first discovered by Berliner in 1915. The mechanism of action of *Bt* endotoxin (Cry) involves binding to specific receptors located on the gut epithelial layer of invertebrates under alkaline conditions (Lepidoptera), followed by pore formation and cell lysis [6,7].

Wax moth *Galleria mellonella* L. is one of the most commonly used models for toxicological investigations [21,22,23,24], including analysis of the antibacterial action of drugs [25] and in vivo assessment of pathogenicity of various microorganisms [26,27,28]. Wax moth larvae develop toxicosis in the midgut, which is accompanied by changes in the microbiota, digestion activity, and defense systems [11,12]. The moth microbiota is mainly composed of bacteria belonging to the phylum Firmicutes; these typically are different species of Gram-positive Enterococci (*E. faecalis*, *E. mundtii*, *E. innessi*, etc.) [12,28,29,30,31,32]. Enterococci can produce a variety of antimicrobial peptides that help suppress gut infections [4,33]; gut Firmicutes are known to have a general ability to degrade insecticides [20,34].

The role of the microbiota in the formation of insects’ resistance to *Bt* still remains open. The symbiotic gut bacteria are able to act as a protective barrier to *Bt* [35,36], synergistically or additively interact with pathogen [19,37], change their status from commensal to pathogenic by penetration of the insects’ hemocoel [38], or are not required at all for *Bt* pathogenicity [39].

We hypothesize that changes in the ecological conditions (e.g., permanently feeding a diet with an antibiotic for several generations) will lead to host adaptation due to the alterations in both sensitivity of the host to *Bt* in the gut microbiota community and in the physiological state of the insects.

In this study, wax moth larvae were fed a diet with amikacin (a broad-spectrum antibiotic). In the eighteenth filial generation, we analyzed the midgut microbiota to assess the activity of proteolytic and antioxidant enzymes and identified the midgut tissue structure using ultra-thin section electron microscopy. Furthermore, we compared some vital signs (pupa weight and number of eggs in the oviposition), as well as estimated the sensitivity of *G. mellonella* larvae to the entomopathogenic bacteria *Bacillus thuringiensis*.

## 2. Materials and Methods

### 2.1. Insects and Experimental Design

The laboratory lines of wax moth *Galleria mellonella* L. were cultivated at a constant temperature of 28 °C and 20% relative humidity under a 12 h photoperiod and fed an artificial diet [40]. Some newborn wax moth larvae were fed an artificial diet with antibiotic solution (a diet with antibiotic). The semi-broad-spectrum synthetic antibiotic, amikacin (Sintez, Russia), was used in the experiments. Prior to the experiment, the sensitivity of *Enterococcus faecalis* (GC1) to amikacin was evaluated using a standard in vitro disk diffusion test. Specifically, 10 mm paper disks soaked in an antibiotic solution (A—0.935, B—1.87, and C—3.75 mg/L) were plated. *E. faecalis* (GC1) from the collection of the Institute of Systematics and Ecology of Animals, Siberian Branch of the Russian Academy of Sciences (ISEA SB RAS) was used. No bacterial growth inhibition was observed after 48 h incubation at 28 °C (see Supplementary Figure S1A–C). Amikacin was dissolved in sterile water (volume specified in the recipe) and added at the end of preparing 1000 g of artificial diet. The final amikacin concentration was 1.5 × 10^−2^%. The wax moth line was continuously cultivated on a diet with an antibiotic for 18 generations. In parallel, the wax moth line was maintained on an antibiotic-free diet (the typical diet). In the eighteenth generation of *G. mellonella* larvae, both lines (IV instar, at least 8 h post molting) were cryo-anesthetized at −4 °C, followed by surface sterilization and midgut dissection for further analysis.

### 2.2. 16S rDNA Metabarcoding

Two lines of wax moth larvae fed the typical diet and the diet with antibiotic were treated with 0.05% chlorhexidine. Midguts with contents were isolated, homogenized, and frozen in liquid nitrogen (four samples per diet variant, five midguts per sample).

The DNeasy PowerSoil Kit (Qiagen, Hilden, Germany) was used for total DNA extraction according to the manufacturer’s instructions; bead beating was performed using TissueLyser II (Qiagen, Hilden, Germany) for 10 min at 30 Hz. Agarose gel electrophoresis was carried out to assess the quality of the extracted DNA; no further DNA purification was needed.

The V3–V4 region of the 16S rRNA genes was amplified with the primer pair 343F (5′-CTCCTACGGRRSGCAGCAG-3′) and 806R (5′-GGACTACNVGGGTWTCTAAT-3′) combined with Illumina (San Diego, CA, USA) adapter sequences [41]. PCR amplification was performed according to the procedure described earlier [42]. A total of 200 ng of the PCR product from each sample was pooled together and purified using a MinElute Gel Extraction Kit (Qiagen, Hilden, Germany). The obtained amplicon libraries were sequenced with 2 × 300 bp paired-ends reagents on a MiSeq platform (Illumina) at the Genomics Core Facility, SB RAS (ICBFM SB RAS, Novosibirsk, Russia). The read data reported in this study were submitted to the NCBI Short Read Archive under BioProject accession number PRJNA980557.

Raw sequences were analyzed via the UPARSE pipeline [43] using the Usearch v11.0.667 software. The UPARSE pipeline included merging of paired reads, read quality filtering, length trimming, merging of identical reads (dereplication), discarding singleton reads, removing chimeras, and operational taxonomic unit (OTU) clustering using the UPARSE-OTU algorithm.

The OTU sequences were assigned a taxonomy using the SINTAX [44] and 16S RDP training set v18 as a reference [45]. Alpha diversity metrics were calculated using the Usearch software. Principal component analysis (PCA) was performed for the data using Python’s scikit-learn package [46]. The Mann–Whitney U test was performed using the Python scientific computing library, SciPy (v.1.5.1) [47].

### 2.3. Changes in CFU Counts in the Midgut of G. mellonella F18

The surface of wax moth larvae was sterilized with 0.05% chlorhexidine; the midguts with the contents were then isolated, mechanically homogenized, and suspended in 1 mL of 150 mM sterile saline solution (SS). Aliquots (100 µL) from diluted suspensions at 10^−2^ were inoculated onto the surface of bile esculine azide agar (Himedia, India). Bacterial colonies were counted after 48 h of incubation at 28 °C. Fourteen samples from each group were used for analysis.

### 2.4. Identification of Bacteria and Their In Vitro Sensitivity to the Antibiotic

Bacterial colonies were isolated into a pure culture by passaging three times on the aforementioned medium under the same conditions (28 °C). Pure cultures were identified using 16S rRNA sequencing according to the procedure described previously [48]. The obtained 16S sequences for isolates were deposited into the Genbank (Nos. OR018313–OR018329).

In order to determine the sensitivity to amikacin, a symbiotic bacteria isolate was incubated with amikacin. An overnight culture of bacteria (20 μL, D_600_ = 1.0) was added to 1 mL of nutrient broth (pH 8.4; Himedia, India) with amikacin (pre-filtered 0.45 nm syringe filter). The final amikacin concentrations in the broth with bacteria were 37.5, 75, and 150 mg/L. Sterile SS was introduced into the broth as a control. After 24 h of cultivation (at 28 °C) of bacteria, optical density at λ = 600 nm (D_600_) was measured (Multiskan Ascent, Thermo Sci., Carlsbad, CA, USA). Three replicates were used for all the bacterial isolates and amikacin concentrations.

### 2.5. Sample Preparation and Enzyme Activity Measurements

Midgut tissues were prepared on ice in phosphate-buffered saline (PBS; pH 7.2). Larval midguts were dissected; their contents were removed and twice rinsed with PBS. The tissues were collected into tubes containing 120 µL of ice-cold PBS supplemented with 0.1 mM *N*-phenylthiourea (PTU). The midguts were suspended using a Bandelin ultrasonic homogenizer (Germany) for 3 s (1 cycle). The supernatant (10,000× *g*, 5 min, 4 °C) was used to spectrophotometrically measure enzyme activity.

The activity of alkaline proteases was measured using the method proposed by Elpidina et al. [49] and Gatehouse et al. [50]. The sample (8 µL) and 500 µL of 5 mM Tris-HCl buffer (pH 8.0) supplemented with 0.25% azocasein (Sigma-Aldrich, Darmstadt, Germany) were mixed and incubated at 24 °C for 40 min. The reaction was stopped by adding 250 µL of 1.1 M C_2_HCl_3_O_2_ (trichloroacetic acid, TCA) and immediately cooled down on ice for 10 min. The supernatant (10,000× *g*, 5 min, 4 °C) was measured at λ = 366 nm. Twenty samples (one sample = two midguts) were used for each variant.

The procedure proposed by Anson [51] and modified by Noskov et al. [52] was used to measure the acidic protease activity. Briefly, 250 µL of 0.3% hemoglobin (BD, France) solution in PBS (pH 6.0) and the sample (30 µL) were incubated at 27 °C for 20 min. The reaction was stopped by adding 250 µL of 0.3 M TCA. Supernatant (10,000× *g*, 5 min) was measured at λ = 280 nm. Twenty samples (one sample = two midguts) were used for each variant.

The activity of glutathione-*S*-transferases (GSTs) was measured colorimetrically according to formation of 5-(2.4-dinitrophenyl)-glutathione based on the method proposed by Habig et al. [53]. Here, 200 µL of ice-cold substrate [49.4 mM C_6_H_3_ClN_2_O_4_ dissolved in acetone and 0.98 mM glutathione in PBS] was added to the 5 µL sample. The reaction mixture was incubated at 28 °C for 15 min, and optical density at λ = 410 nm was measured. Twenty samples (one sample = two midguts) were used for each variant.

Peroxidase activity was measured using 4-aminoantipyrine as a substrate according to the procedure proposed by Nicell and Wright [54] with some modification. The samples (20 µL) were mixed with 100 µL of the reaction mixture [0.17 M C_2_H_5_OH, 1.7 mM H_2_O_2_ and 2.5 mM 4-aminoantipyrine dissolved in PBS (pH 7.2)]. After incubation (4 min at 25 °C in the dark), the mixture was incubated for measuring optical density at λ = 510 nm. Fourteen samples (one sample = two midguts) were used for each variant.

Catalase activity was assayed by measuring optical density at λ = 240 nm according to the rate of hydrogen peroxide decomposition [55]. Briefly, 195 µL of the reaction mixture [1.17 mM H_2_O_2_ in PBS (pH 7.0)] and 5 µL samples were mixed, and optical density was measured (after 60 s at 25 °C). Twenty samples (one sample = two midguts) were used for each variant.

The protein concentration was determined using the method described by Bradford [56]. Bovine serum albumin was used to generate the standard curve. Enzyme activity was measured in optical density units (∆A) of the incubation reaction mixture per 1 min (or 1 s for catalase) and 1 mg of protein.

### 2.6. pH of the Midgut Contents and Electron Microscopy of the Midguts

Larval midguts were isolated on ice-cold SS. The contents of the midgut were removed and collected in tubes containing 1 mL of deionized water. pH of the midgut contents of both wax moth lines was determined. At least 10 samples were used for each variant.

Midgut tissues were rinsed thrice in sodium cacodylate buffer. The midguts were placed into a fixative solution [2% glutaraldehyde in 0.1 M sodium cacodylate buffer (pH 7.2)] and maintained at 4 °C for 24 h. The midgut samples were stained, dehydrated, and sectioned according to the procedure described by Polenogova et al. [57].

### 2.7. Vital Signs

The pupa weight (200 individuals from each group) and the number of eggs in the oviposition (40 oviposition samples for each group) of *G. mellonella* F18 after cultivation on a diet with antibiotic and a typical diet were recorded.

### 2.8. Preparing Bacteria and Performing Bioassay

Entomopathogenic bacteria *B. thuringiensis* var. *galleriae* 69-6 (*Bt*) from the collection of the Institute of Systematics and Ecology of Animals (ISEA) SB RAS was used for in vitro assessment of sensitivity to amikacin (as described in Section 2.1. Insects and experiment design) and for treatment. *E. mundtii* 2521 and *E. innessi* 1721 strains were used after their isolation and identification during the treatment study. All the bacteria were cultivated on nutrient agar (pH 7.2; Himedia, India) at 28 °C for 6 days (for *Bt*) and 24 h (for *E. mundtii* 2521 and *E. innessi* 1721). Bacterial suspension was prepared in SS and pre-rinsed twice with SS (6000× *g* for 10 min). The bacterial titer was determined using a Neubauer hemocytometer. The entomopathogenic bacterial titer of *Bt* was 2 × 10^8^ spores and crystals/mL; the titer of both symbiotic bacteria *E. mundtii* 2521 and *E. innessi* 1721 was 7 × 10^7^ cells/mL. The spore:crystal ratio was 1:1 in a microbiological smear dyed with eosin. In the control group, 1 mL of sterile SS was added. Wax moth larvae of the eighteenth generation up to the IV instar (4–6 h post molting) were kept without food for 2 h before the experiment and then fed 3 g of artificial diet with 1 mL suspension of bacteria. Four replicates were used for each variant (1 replicate = 30 larvae). Survival was assessed over 7 days. The following variants were used to perform bioassays with both insect lines (the typical diet and diet with antibiotic): control, *Bt*, *E. mundtii* 2521, *Bt* + *E. mundtii* 2521, *E. innessi* 1721, and *Bt* + *E. innessi* 1721.

### 2.9. Statistics

Statistics and data visualization were performed using STATISTICA 8.0, Past3, and GraphPad Prism 5. The normality of the data distribution was checked using the Shapiro–Wilk W test. Normally distributed data (*p* > 0.05) were subjected to *t*-tests. Non-normally distributed data (*p* < 0.05) were subjected to Mann–Whitney U-tests. Differences in the mortality rate were analyzed using the Kaplan–Meier log-rank test (Sigma-Stat 3, Systat Software Inc., Tulsa, OK, USA).

## 3. Results

### 3.1. Bacteriobiome

The samples were collected after cultivating eighteen generations of *G. mellonella* larvae reared on the standard diet and a diet containing amikacin. Metabarcoding sequencing of the 16S rDNA was performed. The final dataset contained 115 OTUs and 236,085 reads (29,492 ± 1175 reads per sample, see File S1). All the rarefaction curves followed a trend of approaching the saturation plateau, which indicated a reasonable volume of reads (see Supplementary Figure S2). Based on 16S rDNA metabarcoding, three main phyla, including Firmicutes, Proteobacteria, and Actinobacteria (identity > 99%), were identified in the bacterial community in the midgut of moth larvae.

Cultivation of 18 generations of the wax moth fed an antibiotic significantly increased the number of OTUs as well as bacterial diversity (Shannon_10) and richness (Chao1) indices; however, only the Chao1 index was significant compared to the wax moths receiving a typical diet (Mann–Whitney U-test, *p* = 0.03) (Table 1).

**Figure 1:**
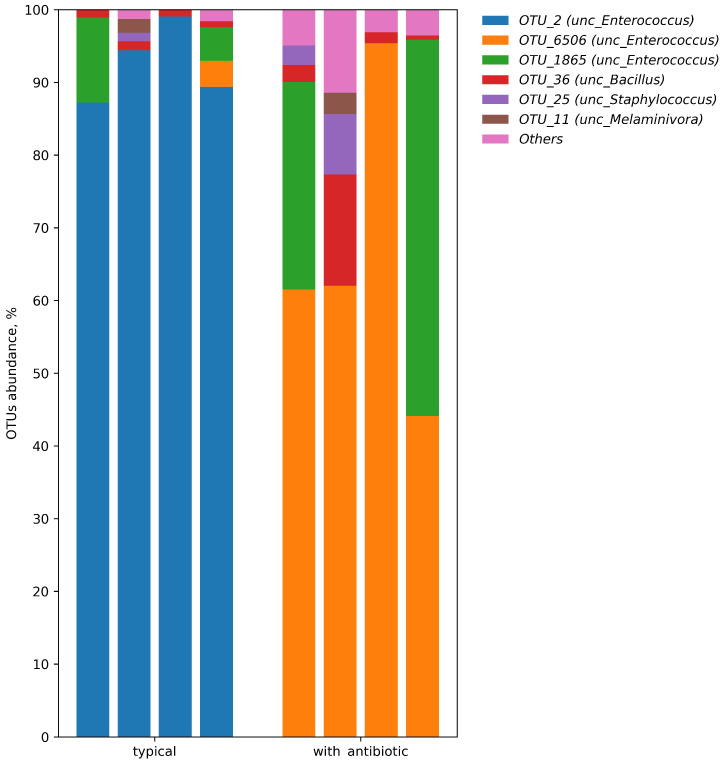
The main operational taxonomic units (OTUs) of the midgut microbiota composition of *G. mellonella* larvae (F18 generation) after cultivation on a typical diet and a diet supplemented with an antibiotic (a final concentration of amikacin of 1.5 × 10^−2^%). Both variants included four samples (one sample = five midguts).

**Figure 2:**
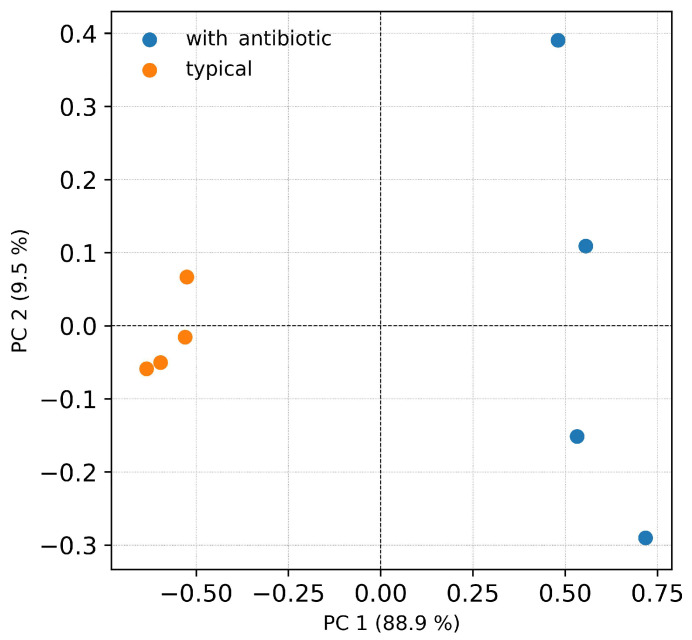
Principal component analysis of the OTU level of the midgut microbiota of *G. mellonella* larvae (F18 generation) after cultivation on a typical diet and a diet supplemented with an antibiotic (a final concentration of amikacin of 1.5 × 10^−2^%). Both variants included four samples (one sample = five midguts).

The abundance of OTUs in Firmicutes samples ranged from 86 to 100% (Figure 1). In the 18th generation of wax moths, an antibiotic diet significantly reduced their abundance, but only as a trend (Mann–Whitney U-test, *p* = 0.054 (Table 2). Meanwhile, we recorded an increased abundance of OTUs of Actinobacteria and Proteobacteria compared to that in wax moths fed a typical diet, but only as a trend (*p* = 0.06). Significant changes in the relative abundance of bacteria within the Firmicutes phylum referred to two OTUs of the genus Enterococcus (class Bacilli). Specifically, the abundance of *E. faecalis* (OTU_2) decreased more than 90 fold, whereas the abundance of *E. mundtii* (OTU_6506) increased 73-fold compared with the typical diet (*p* = 0.02 and *p* = 0.03, respectively). The five-fold increase in the relative abundance of *E. xiangfangensis*/*E. devriesei* (OTU_1865) was not significant (*p* = 0.19). The first two principal components from the PCA accounted for over 98% of the total variation in the original microbial community fed different diets (Figure 2).

### 3.2. CFU Counts and Identification of Bacteria

**Figure 3:**
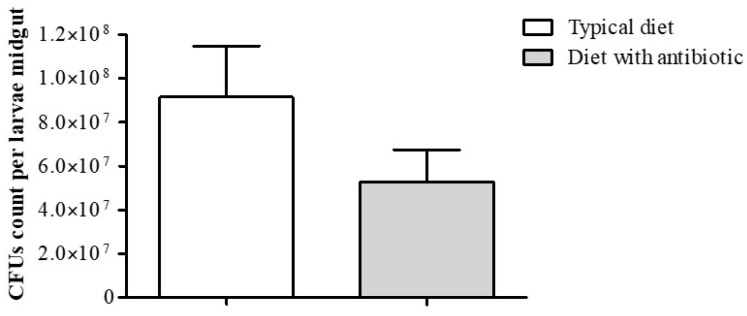
Changes in the CFU counts of Enterococci in the *G. mellonella* larvae (F18 generation) midguts after cultivation on a typical diet and a diet supplemented with an antibiotic (the final concentration of amikacin was 1.5 × 10^−^^2^%). Both variants included 14 samples (one sample = one midgut).

In order to determine how the midgut bacteria influence the wax moth’s adaptation to a diet supplemented with antibiotic, the larval midguts with their contents were plated onto microbiological medium. Analysis of the CFU count showed that the number of cultivated bacteria in insects fed a diet that contained the antibiotic was slightly decreased (×1.7), but the result was not significant (Mann–Whitney U-test, *p* = 0.19) (Figure 3).

The predominant bacterial colonies present in the microbiota of both groups of larvae were isolated and identified. The results showed that all the identified bacteria belonged to the genus Enterococcus. Specifically, 16S rRNA gene-based identification of these cultures showed 100% identity with *E. faecalis*, *E. mundtii*, and *E. innesii* (Table 3). The midgut microbiota of *G. mellonella* larvae (F18 generation) cultured on a diet supplemented with an antibiotic included *E. mundtii* and *E. innesii* bacteria. Meanwhile, *E. faecalis* prevailed in the midgut of larvae raised on a typical diet. This result is consistent with the findings of metabarcoding taxonomic identification of the bacterial community in the midgut of the wax moth larvae (Figure 1).

### 3.3. Enzymatic Activities in Midgut Tissues

**Figure 4:**
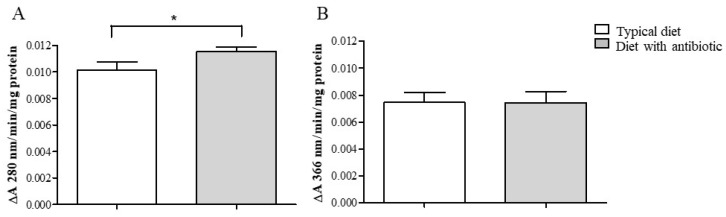
Activity of acidic (**A**) and alkaline (**B**) proteases of midgut tissues of *G. mellonella* (F18 generation) after cultivation on a typical diet and a diet supplemented with an antibiotic (the final amikacin concentration was 1.5 × 10^−^^2^%). *—significant intergroup differences (for acidic proteases: Mann–Whitney U-test, *p* = 0.016).

In the midgut tissues of the 18th generation *G. mellonella* larvae, the antibiotic diet slightly (1.14 fold) but statistically significantly increased acidic protease activity compared to that in the insects fed a typical diet (Mann–Whitney U test, *p* = 0.016) (Figure 4A). The level of alkaline protease activity in midgut tissues did not differ between the groups (*t*-test, *p* = 0.98) (Figure 4B).

**Figure 5:**
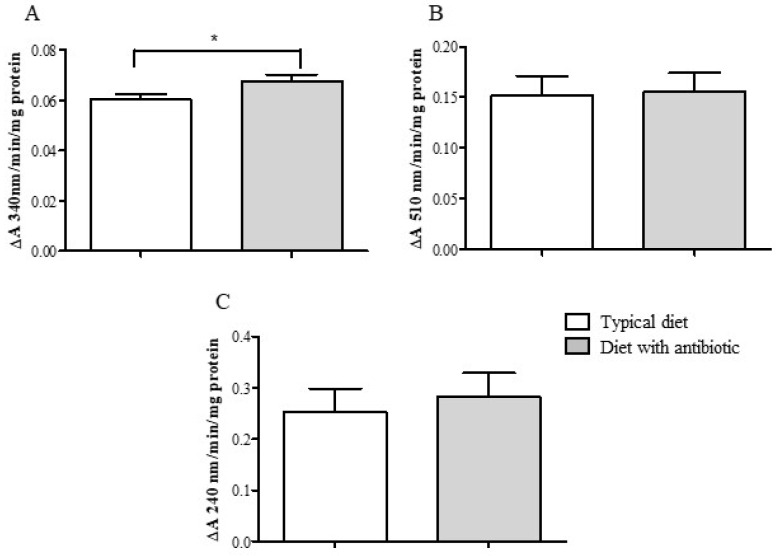
Antioxidant enzyme activity of (**A**) glutathione-*S*-transferase, (**B**) peroxidase, and (**C**) catalase of the midgut tissues of *G. mellonella* larvae (F18 generation) after cultivation on a typical diet and a diet supplemented with an antibiotic (the final amikacin concentration was 1.5 × 10^−^^2^%). *—significant intergroup differences (*t*-test, *p* = 0.03).

Cultivation of wax moth with antibiotic resulted in a minor but valid 1.12-fold increase in GST activity (*t*-test, *p* = 0.03, compared with the typical diet) (Figure 5A). Peroxidase and catalase activities did not differ significantly between the wax moth lines fed different diets (*p* = 0.9 and *p* = 1.0, respectively) (Figure 5B,C).

### 3.4. pH of the Midgut Contents and Ultra-thin Sections

**Figure 6:**
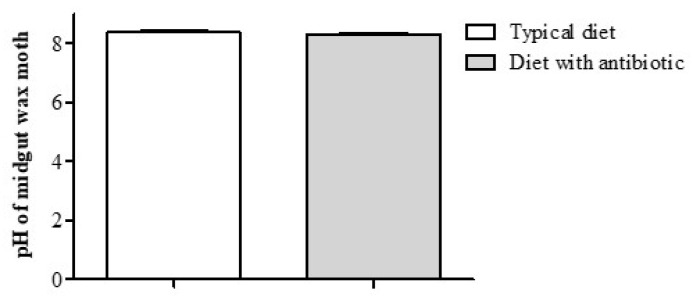
pH of the midgut contents of the *G. mellonella* larvae (F18 generation) after cultivation on a typical diet and a diet supplemented with antibiotic (the final amikacin concentration was 1.5 × 10^−^^2^%) (*t*-test, *p* > 0.05). pH values are shown as the mean ± SE (n = 10).

No changes in pH in the midgut contents of the F18 moth larvae were observed (*t*-test, *p* = 0.34, compared with the typical diet) (Figure 6).

**Figure 7:**
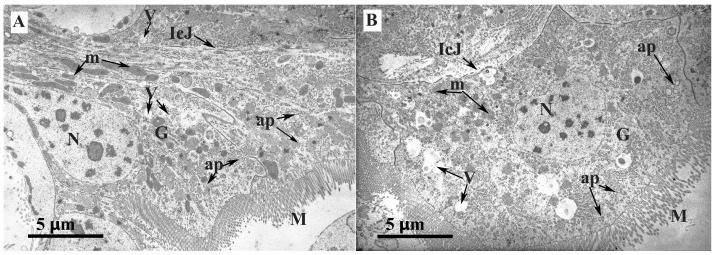
Ultra-thin sections of the midgut larvae of *G. mellonella* (F18 generation) after cultivation on a typical diet and a diet supplemented with an antibiotic (the final amikacin concentration was 1.5 × 10^−^^2^%). Here, 4000× magnification is presented for both variants: (**A**) typical diet samples; (**B**) diet with antibiotic. **M**—microvilli; **ap**—apocrine bubbles; **m**—mitochondria; **N**—nucleus; **V**—vesicles; **IcJ**—intercellular junctions; **G—**Golgi apparatus.

An analysis of ultra-thin intestinal sections of both wax mole lines showed no morphological differences in the structure of midgut epithelial cells (Figure 7).

### 3.5. Vital Signs of the F18 Generation G. mellonella

In the F18 generation, the antibiotic diet significantly reduced (1.14 fold) the weight of wax moth pupae (*t*-test, *p* < 0.001, compared with the typical diet) (Table 4). No intergroup differences in the fertility rate of eggs in the oviposition were revealed.

### 3.6. Influence of Amikacin on Bt Growth

**Figure 8:**
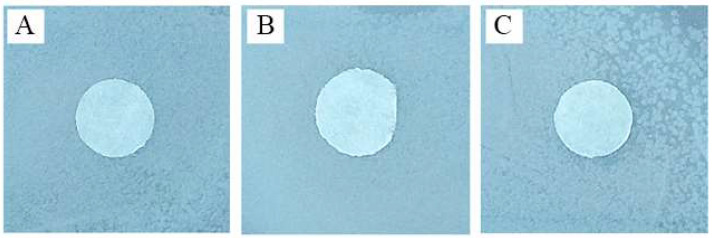
The sensitivity of *B. thuringiensis* var. *galleria* 69-6 to amikacin (Synthesis, Russia) was determined in vitro using diffusion disks. The effect of amikacin on bacterial growth was assessed by plating 10 mm paper disks soaked in the following concentrations of antibiotic solution onto freshly plated bacterial culture lawns: (**A**) 0.935, (**B**) 1.87, and (**C**) 3.75 mg/L. The zones of inhibition were measured after 48-h incubation at 28 °C. The assays were performed in four replicates.

Before assaying the sensitivity of *G. mellonella* F18 larvae to *Bt*, we assessed the effect of amikacin on the bacteria. In vitro, *Bt* showed resistance to all amikacin doses (Figure 8).

### 3.7. Sensitivity of G. mellonella F18 to Bt

**Figure 9:**
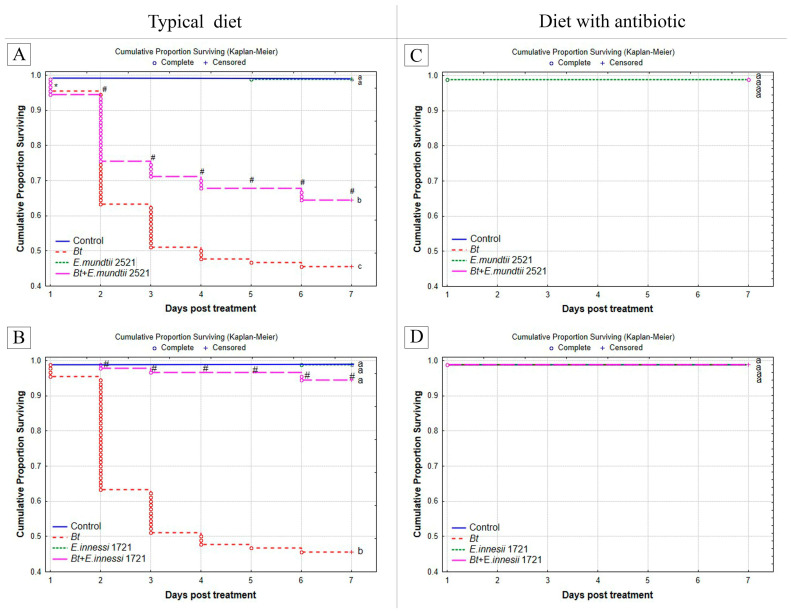
Survival of the F18 generation of *G. mellonella* after culturing on (**A**,**B**) the typical diet and (**C**,**D**) the diet with antibiotic (final concentration of amikacin, 1.5 × 10^−^^2^%) after treatment with entomopathogenic bacteria *Bacillus thuringiensis* var. *galleriae* (2 × 10^8^ spores and crystals/mL) and symbiotic bacteria (7 × 10^7^ cells/mL). (**A**,**C**) *Enterococcus mundtii* 2521, (**B**,**D**) *Enterococcus innessi* 1721. Before the experiment, an artificial diet with saline solution was prepared for the larvae in the control group. Larvae were maintained without food for 2 h. The artificial diet was previously sterilized by gamma radiation. All the variants included four replicates (one replicate = 30 larvae). Different letters (a–c) show significant intergroup differences in the survival of wax moths as estimated using the log rank test (χ^2^ > 5.6, df = 1, *p* < 0.001, compared to the wax moth line reared on a typical diet). Asterisks indicate additive effects (χ^2^ > 3.84, *p* < 0.05); the hash mark indicates antagonistic effects (χ^2^ > 5.6, *p* < 0.001).

Inoculation of *G. mellonella* larvae (F18) fed a diet supplemented with an antibiotic by entomopathogenic bacteria *Bt* did not lead to insect death; their survival rate was 100% (Figure 9C,D). Meanwhile, in larvae reared on a typical diet, *Bt* feeding resulted in the development of bacterial infection, and larval survival was 50% (*p* < 0.001, compared with control group) (Figure 9A,B). Next, we analyzed the role of the isolated symbiotic enterococci (*E. mundtii* 2521 and *E. innessi* 1721) on the susceptibility of larvae cultivated on a typical diet and diet with antibiotic. The separete feeding of enterococci *E. mundtii* 2521 and *E. innessi* 1721 did not lead to the development of bacterial infection. However, inoculation of *G. mellonella* larvae fed a typical diet with both enterococci in combination with *Bt* significantly reduced larval mortality due to additive or antagonistic effects. Differences in the mortality dynamics after the exposure to *Bt* and *Bt* + *E. mundtii* 2521 were significant. On day 1, the effect was additive (log rank test: χ2 = 0.2, df = 1, *p* < 0.05). On days 2–7 post-treatment, the survival of wax moth was increased by the antagonistic effect of both enterococci (1.6 fold for *E. mundtii* 2521, χ2 > 5.6, *p* < 0.001; ninefold for *E. innessi* 1721, χ2 > 45.1, *p* < 0.001, compared with inoculation with *Bt* only; Figure 9A,B). Inoculation of *G. mellonella* (F18) larvae fed the antibiotic diet with enterococci only and in combination with *Bt* did not cause insect death (Figure 9C,D); in the control groups reared on both types of diets, insect survival was 100%.

## 4. Discussion

We showed that long-term cultivation of *G. mellonella* on an artificial diet significantly with a broad-spectrum antibiotic (amikacin) significantly alters the midgut microbiota composition, affects activity of digestive and antioxidant enzymes in it, and reduces pupa weight. We hypothesize that these effects were due to the change in the microbial community and “separation” of amikacin-resistant *E. mundtii*, promoting active digestion and protecting the epithelial layer of the midgut against various toxins, which indicates the adaptation of the wax moth to the antibiotic.

We observed a substantial increase in the number of OTUs, bacterial diversity, and richness in the midgut larvae reared on an amikacin diet; however, richness was the only parameter for which a significant difference was observed compared to the typical diet. Unexpectedly, we observed a 73-fold increase in the abundance of the minor *E. mundtii* after long-term cultivation on a diet with an antibiotic. Other researchers have mentioned in their studies that rearing of *Drosophila nigrosparsa* on a diet with tetracycline for three generations was not accompanied by changes in the alpha diversity of the gut microbiota, although the abundance of *Lactobacillus* (Firmicutes) increased [5].

Microbiological analysis of the midguts of wax moth larvae revealed that CFUs of Firmicutes were slightly decreased in the group of insects reared on a diet supplemented with amikacin. Moreover, identification of individual isolates showed that these data were consistent with the results of metabarcoding analysis and that bacteria belonged to different *Enterococcus* species. It is noteworthy that a significant proportion of the identified bacteria were *E. innessi*, which can potentially be explained by the presence of bacterial colonies of different morphotypes in the midgut of the wax moth. The phenomenon of increased counts of enterococci is fairly common during the development of various toxicoses in insects [8,9,12]. *E. faecalis* are tolerant to aminoglycosides [58]. In our experiments, we also did not observe amikacin causing significant growth inhibition at a biologically relevant level (see Supplementary Figures S1A–C and S3). Although it is known that the results of in vitro and in vivo tests may differ, and it is possible that the potential resistance of *E. mundtii* to the antibiotic reduced the competitiveness of *E. faecalis*. In general, the importance of enterococci for the host can be quite broad and may include both their probiotic role [2,59,60,61] and their ability to produce antimicrobial peptides necessary to suppress the development of various infections [33]. However, it should be noted that studies addressing the effect of antibiotics on the insect microbiota were performed using insects up to the 6th generation (depending on a certain study) (*Drosophila nigrosparsa*) [5,9].

It is most likely that enterococci (*E. mundtii* in this study) may be crucial for adaptation to antibiotics not only for the microbiota, but also for the wax moth per se. The antimicrobial substances synthesized by the microbiota cause microflora depletion and the emergence of microorganisms resistant to these substances, as well as alter the physiological processes in the host [22,62]. *E. mundtii* frequently isolated from the guts of various insects acts as a probiotic in the gut of mill moth *Ephestia kuehniella* [59]; it affects the resistance to organophosphate insecticides in the silkworm *Bombyx mori* [2,60] or antagonizes the action of entomopathogenic bacteria in the beetle *Tribolium castaneum* [59]. Meanwhile, the xenobiotic properties and toxic effects of antibiotics could alter both the immune reactions and enzymatic activities in various organs and tissues. The effect of aminoglycosides, including amikacin, on tissues or organs of the hosts has been reported mostly for vertebrates. In particular, neuro- and nephrotoxic effects have been demonstrated for amikacin. Reduced activity of antioxidants, such as superoxide dismutase (SOD), catalase, glutathione-*S*-transferase (GSH), and glutathione peroxidase (GPx), along with activation of necrosis-like and apoptotic death of the cochlear tissue cells, was observed in guinea pigs treated with amikacin [63]. Similar effects have also been detected in tissue cultures treated with gentamicin (an aminoglycoside antibiotic) [64]. Studies performed on insects were predominately based on the data obtained from one, or six at most, generations reared on an antibiotic diet. Thus, reduced activity of antioxidant enzymes and transaminases was demonstrated for the midgut of the *G. mellonella* larvae fed streptomycin (first generation) [65]. Similar results were obtained on *Drosophila melanogaster*, indicating that exposure to streptomycin reduces activity of antioxidant enzymes [66]. In our experiments, GST activity was increased, while no differences in the activities of other antioxidants (catalase and peroxidase) in the wax moth midgut (F18) reared on different diets were detected. GST often acts not only as an antioxidant, but it can also help detoxify toxic products that accumulate in tissues during destructive processes [67]. In our experiments, we selected an antibiotic dose that had no harmful effect on the insects’ gut cells. This was confirmed by the absence of noticeable differences between the electron microscopic sections of the midgut tissues of the larvae fed the amikacin diet versus the control diet. The absence of significant differences in the activities of antioxidant enzymes of wax moth larvae are likely indicative of the formation of adaptive mechanisms in insects after long-term exposure to an antibiotic.

Certain environmental conditions are required for enzymes, and proteases in particular, to function successfully. For example, serine proteases are active under alkaline conditions, while cysteine proteases are active under acidic conditions. Changes in the microbiota can alter pH of the gut lumen. Thus, the microbiota of the western corn rootworm *Diabrotica virgifera virgifera* contributes to the survival of the host when its food plant is changed (switching to soybeans), and this change is accompanied by an increase in cysteine protease activity in the host [68]. We hypothesize that the increase in *E. mundtii* density in the wax moth midgut increases lactic acid production, thus changing the pH of the gut contents. It is known that lactic acid produced by enterococci can contribute to food digestibility [61] and microbiota regulation [69] and positively affects the viability and reproduction of individuals. However, our measurements of the pH values in the midguts revealed no difference between the diets.

An increased activity level of proteolytic enzymes often results from starvation and/or ongoing destructive processes in tissues during toxicosis. For phytophages, physiological adaptation to xenobiotics is usually accompanied by increased activity of digestive enzymes, which helps reduce the toxic effects of xenobiotics on tissues [70,71,72,73]. Any changes in protease activity or their composition can affect the sensitivity of insects to insecticides and/or entomopathogens [73,74]. Insects are able to use a variety of strategies to minimize the effects of a non-preferred diet. These include the overproduction of existing digestive proteases, expression of inhibitory insensitive digestive proteases [75], and “switching” between several gene copies (proteases, serine, cysteine, and aminopeptidase in particular) [76]. In our case, increased activity of acidic proteases in the midgut tissue at constant pH, as well as in our previous study for the F10 generation of wax moth, mostly attests to the toxic effect of the amikacin and adaptation due to elevated activity of digestive enzymes [77].

Invertebrates can actively use antimicrobial peptides synthesized by symbiotic microorganisms to suppress the development of gut infections [4,33]. We observed no deaths of the wax moth larvae (F18) reared on a diet supplemented with an amikacin after exposure to the entomopathogenic bacteria *B. thuringiensis*. In addition, in vitro tests revealed no antagonistic interactions between the microbiota predominant in the wax moth (enterococci (*E. faecalis* N121 and N1021, *E. mundtii* A2521, *E. innesii* A1721)) and the entomopathogenic bacteria *B. thuringiensis* (see Supplementary Figure S4). We also found no growth inhibition of *B. thuringiensis* in in vitro tests with amikacin discs (0.935, 1.87, and 3.75 mg/L) (Figure 8). However, inoculation with enterococci isolated from midgut of wax moth in combination with *Bt* significantly suppressed the development of infection. This effect may be due to inhibition of *Bt* spores and crystals in vivo by the antibiotic remaining in larval tissues. We performed an additional experiment in which wax moths up to the 17th generation maintained on a diet with antibiotic were transplanted in the 18th generation to a gamma-sterilized diet without the addition of amikacin, starting with newborn larvae, to exclude possible inhibition of *Bt* by amikacin. Oral infection of these IV instar larvae with *Bt* (2 × 10^8^ spores and crystals/mL) did not result in the development of bacterial infection, and insect survival was 100% (see Supplementary Figure S5). On the one hand, increased activity of proteolytic enzymes as the host uses the microbiota proteases is expected to increase the virulence of the gut pathogens. Thus, trypsin proteases belonging to the serine (or alkaline) group of proteolytic enzymes are required for solubilization and activation of *B. thuringiensis* Cry-toxins under alkaline midgut conditions in Lepidoptera [78,79]. At least two secreted proteases produced by *E. faecalis*, which is dominant in the midgut of wax moth, were observed: metalloproteinase gelatinase (GelE) and serine protease (SprE) [80]. It is possible that they are involved in limited proteolysis of the *B. thuringiensis* Cry toxin protein. On the other hand, although no virulence factors inherent in other enterococci were identified for *E. mundtii* [81], it contains the collagen adhesin genes of *E. faecium* (*scm*) [82] and the mundticin munST4SA gene (a bacteriocin active against various bacteria: *E. faecalis*, *Streptococcus pneumoniae*, *Staphylococcus aureus*, and *Listeria monocytogenes*) [83]. Furthermore, in *Spodoptera littoralis*, increased bacterial density and expression of the genes controlling adherence of *E. mundtii* to the midgut epithelium leads to the formation of biofilms promoting pathogen suppression and protecting gut cells against oxidative stress [84]. Grau et al. [59] investigated the probiotic effect of *E. mundtii* for the red flour beetle *Tribolium castaneum* infected with *B. thuringiensis*. They found that the survival of larvae pre-fed with the supernatant of *E. mundtii* bacteria was significantly increased, but the lifespan and fertility of the insects were simultaneously reduced. These findings are in good agreement with our results. Although we have not monitored the lifespan of the wax moth in our experiments, pupa weight was significantly decreased. For some *E. innesii* strains isolated from *G. mellonella*, the absence of non-virulence genes or antibiotic resistance genes and their importance in inhibiting metamorphic transformation of the wax moth are known [32,85]. Moreover, Kong et al. showed that repeated inoculation of *E. innessi* onto *G. mellonella* larvae induced expression of antimicrobial peptide genes encoding gallerimycin, cecropin, and IMPI (insect metalloprotease inhibitor), and increased expression of these genes persisted even 48 h after inoculation [85].

## 5. Conclusions

We suggest that the increase in *E. mundtii* or *E. innessi* density in the midgut of *G. mellonella* larvae could be an adaptation mechanism of the moth to the altered environmental conditions aimed at protecting the host cells and organs against the effects of toxic substances and pathogens. We found that long-term cultivation (F18) of wax moth reared on a diet with antibiotic (i) significantly (73 fold) increased the density of minor *E. mundtii*; (ii) increased GST activity in the midgut tissues; (iii) increased the activity of acidic proteases in the midgut tissues, while pH in the midgut remained unchanged; and (iv) increased counts of bacteria *E. mundtii* or *E. innessi* in the midgut of *G. mellonella* larvae significantly suppressed the development of bacterial infection caused by *B. thuringiensis*. The present work opens up new prospects for further research into antibiotic-resistant symbiotic microorganisms and their benefits for persistence in the dynamic environment of the insect’s gut, as well as conditions for changing their strategy and manifestation of virulent properties. We believe that one of the tools mediating the adaptation of microorganisms in various communities is the production of secondary metabolites, including those with characteristic antibiotic activity. Due to the synthesis of antimicrobial substances, microorganisms in the microbiota can exist for a long time and maintain a certain localization.

## Figures and Tables

**Table 1:** Diversity characteristics of bacterial communities of the *G. mellonella* F18 generation after cultivation with a typical diet and a diet supplemented with an antibiotic (the final concentration of amikacin was 1.5 × 10^−^^2^%). The results are presented as the median and the 25th and 75th quartile ranges for four replicates.

Diversity	Quantitative Parameters	Diet of the Insect Group		*p*-Value < 0.05 (Mann–Whitney U-Test)
		Typical	with Antibiotic	
Shannon_10	Median	0.14	0.4	0.2
	Lower and upper quartiles	0.11–0.19	0.34–0.51	
Chao1	Median	11.5	25.4	0.03
	Lower and upper quartiles	5.5–18.0	18.88–31.0	

**Table 2:** Changes in the relative abundance of the main phyla and OTUs of the midgut microbiota of *G. mellonella* larvae (F18 generation) after cultivation on a typical diet and a diet supplemented with an antibiotic (a final concentration of amikacin 1.5 × 10^−^^2^%). The results are presented as the median and 75th and 25th quartile ranges for four replicates.

Taxons	Quantitative Parameters	Diet of the Insect Group		*p*-Value < 0.05 (Mann–Whitney U-Test)
		Typical	with Antibiotic	
Firmicutes	Median	99.19	96.44	0.06
	Lower and upper quartiles	98.14–99.94	93.52–97.01	
OTU_2 (*Enterococcus faecalis*)	Median	92.5	0	**0.02**
	Lower and upper quartiles	88.83–95.57	0	
OTU_6506 (*Enterococcus mundtii*)	Median	0.89	65.75	**0.03**
	Lower and upper quartiles	0–0.9	57.16–70.36	
OTU_1865 (*Enterococcus devriesei*/ *E. xiangfangensis*)	Median	4.08	20.07	0.19
	Lower and upper quartiles	0–6.39	0–34.34	
OTU_36 (unc. *Bacillus*)	Median	0.95	1.91	0.34
	Lower and upper quartiles	0–1.08	1.27–5.57	
OTU_25 (unc. *Staphylococcus*)	Median	0	1.33	0.4
	Lower and upper quartiles	0–0.29	0–4.08	
Proteobacteria	Median	0.58	2.37	0.20
	Lower and upper quartiles	0.06–0.61	0.27–2.43	
OTU_11 (unc. *Melaminivora*)	Median	0	0	1.00
	Lower and upper quartiles	0–0.47	0–0.73	
Actinobacteria	Median	0.47	2.00	0.06
	Lower and upper quartiles	0–0.77	1.51–2.32	
Others	Median	0	1.4	0.19
	Lower and upper quartiles	0–0.09	1.29–1.72	

**Table 3:** Putative identification of 16S rRNA (~1400 bp) gene sequences of Enterococci isolated from the *G. mellonella* midgut of the F18 generation using the BLAST technique against procaryotic genomes from GenBank.

Diet of the Insect Group	Isolate Number	Nearest Isolate from GenBank	Identity (%)	OTU Number	GenBank Accession Number
Typical diet	N121	*Enterococcus faecalis* (DACBQW010000003)	100	OTU_2	OR018313
	N221	*Enterococcus innesii* (JAHHEN010000001)	100	OTU_1865	OR018314
	N1021	*Enterococcus faecalis* (DACBQW010000003)	100	OTU_2	OR018315
	N1321	*Enterococcus faecalis* (DACBQW010000003)	99.93	OTU_2	OR018316
	N1721	*Enterococcus faecalis* (DACBQW010000003)	100	OTU_2	OR018317
	N1821	*Enterococcus faecalis* (DACBQW010000003)	100	OTU_2	OR018318
	N2121	*Enterococcus faecalis* (DACBQW010000003)	100	OTU_2	OR018319
Diet with antibiotic	A1121	*Enterococcus innesii* (JAHHEN010000001)	100	OTU_1865	OR018320
	A1621	*Enterococcus mundtii* (WXPA01000019)	100	OTU_6506	OR018321
	A2021	*Enterococcus innesii* (JAHHEN010000001)	100	OTU_1865	OR018322
	A2521	*Enterococcus mundtii* (WXPA01000019)	100	OTU_6506	OR018323
	A121	*Enterococcus innesii* (JAHHEN010000001)	100	OTU_1865	OR018324
	A521	*Enterococcus innesii* (JAHHEN010000001)	100	OTU_1865	OR018325
	A921	*Enterococcus innesii* (JAHHEN010000001)	100	OTU_1865	OR018326
	A1421	*Enterococcus innesii* (JAHHEN010000001)	100	OTU_1865	OR018327
	A1721	*Enterococcus innesii* (JAHHEN010000001)	100	OTU_1865	OR018328
	A2221	*Enterococcus innesii* (JAHHEN010000001)	100	OTU_1865	OR018329

**Table 4:** Vital signs of the *G. mellonella* (F18 generation) after cultivation on a typical diet and a diet supplemented with an antibiotic (the final amikacin concentration was 1.5 × 10^−^^2^%): the pupa weight (200 individuals from each group) and the number of eggs in the oviposition (40 oviposition samples from each group) (mean ± SD).

Vital Signs	Diet of the Insect Group		*p*-Value < 0.001 (*t*-Test)
	Typical	with Antibiotic	
Mass of pupae, mg	0.15 ± 0.0025	0.13 ± 0.0026	0.000
Number of eggs in the oviposition	155.33 ± 13.4	189.65 ± 23.55	0.21

## Data Availability

The metabarcoding read data reported in this study were deposited in GenBank under the study accession No. PRJNA980557. The final dataset data of 16S rRNA metabarcoding are presented in the Electronic Supplementary Material (File S1). The obtained 16S sequences for isolates of bacteria were deposited into the Genbank (Nos. OR018313–OR018329). Other raw data from this study will be provided by the authors upon request, without restrictions.

## Supplementary Materials

The following supporting information can be downloaded at: https://www.mdpi.com/2075-4450/14/11/889/s1. Figure S1. The sensitivity of *Enterococcus faecalis* GC1 [12] to amikacin (Synthesis, Russia) was determined in vitro using diffusion disks. The effect of amikacin on bacterial growth was assessed by plating 10 mm paper disks soaked in an antibiotic solution (A—0.935, B—1.87 and C—3.75 mg/L) on to freshly plated bacterial culture lawns. The inhibition zones were measured after 48 h incubation at 28 °C. The assays were performed in four replicates; Figure S2. Rarefaction analysis of the investigated samples: typical diet (A) and diet with antibiotic (B); Figure S3. Growth of enterococci isolated of *G. mellonella* midgut bacteria (generation F18) after cultivation on typical artificial diet (*Enterococcus faecalis* N121 and *Enterococcus faecalis* N1021) and diet with antibiotic (*Enterococcus innesii* A1721 and *Enterococcus mundtii* A2521). An overnight bacterial culture was incubated in nutrient broth in the presence of an antibiotic (37.5, 75, and 150 mg/L). Sodium chloride solution (SS) was used as a control. The optical density (D_600_) of the broth was evaluated after 24 h of cultivation at 28 °C. Three replications were included for each variant of experiment. *—significant differences compared to SS (*t*-test, *p* = 0.03); Figure S4. Interaction of enterococci isolated from the midgut *G. mellonella* (generation F18) and entomopathogenic bacteria *Bacillus thuringiensis* var. *galleria* 69-6 as assessed by the agar plug diffusion method on nutrient agar (pH 8.4). Petri dishes were incubated at 28 °C. No bacterial growth inhibition was detected after 48 h of incubation at 28° C. Assays were performed in four replicates. File S1 The final dataset contained 115 OTUs and 236,085 reads (29,492 ± 1175 reads per sample).

## References

[1] Maguire R., Kunc M., Hyrsl P., Kavanagh K. (2017). Caffeine administration alters the behavior and development of *Galleria mellonella* larvae. Neurotoxicol. Teratol..

[2] Chen B., Zhang N., Xie S., Zhang X., He J., Muhammad A., Sun C., Lu X., Shao Y. (2020). Gut bacteria of the silkworm *Bombyx mori* facilitate host resistance against the toxic effects of organophosphate insecticides. Environ. Int..

[3] Li Y., Schal C., Pan X., Huang Y., Zhang F. (2020). Effects of antibiotics on the dynamic balance of bacteria and fungi in the gut of the german cockroach. J. Econ. Entomol..

[4] Zhang X., Feng H., He J., Muhammad A., Zhang F., Lu X. (2022). Features and colonization strategies of *Enterococcus faecalis* in the gut of *Bombyx mori*. Front. Microbiol..

[5] Weiland S.O., Detcharoen M., Schlick-Steiner B.C., Steinet F.M. (2022). Analyses of locomotion, wing morphology, and microbiome in *Drosophila nigrosparsa* after recovery from antibiotics. Microbiologyopen.

[6] Blanquart F., Lehtinen S., Lipsitch M., Fraser C. (2018). The evolution of antibiotic resistance in a structured host population. J. R. Soc. Interface.

[7] Larsson D.G.J., Flach C.F. (2021). Antibiotic resistance in the environment. Nat. Rev. Microbiol..

[8] Noman M.S., Shi G., Liu L.J., Li Z.H. (2021). Diversity of bacteria in different life stages and their impact on the development and reproduction of *Zeugodacus tau* (Diptera: Tephritidae). Insect Sci..

[9] Zhang X., Wang X., Guo Z., Liu X., Wang P., Yuan X., Li Y. (2022). Antibiotic treatment reduced the gut microbiota diversity, prolonged the larval development period and lessened adult fecundity of *Grapholita molesta* (Lepidoptera: Tortricidae). Insects.

[10] Jose P.A., Ben-Yosef M., Jurkevitch E., Yuval B. (2019). Symbiotic bacteria affect oviposition behavior in the olive fruit fly *Bactrocera oleae*. J. Insect Physiol..

[11] Kryukov V.Y., Kosman E., Tomilova O., Polenogova O., Rotskaya U., Tyurin M., Alikina T., Yaroslavtseva O., Kabilov M., Glupov V. (2020). Interplay between fungal infection and bacterial associates in the wax moth *Galleria mellonella* under different temperature conditions. J. Fungi.

[12] Polenogova O.V., Kabilov M.R., Tyurin M.V., Rotskaya U.N., Krivopalov A.V., Morozova V.V., Mozhaitseva K., Kryukova N.A., Alikina T., Kryukov V.Y. (2019). Parasitoid envenomation alters the *Galleria mellonella* midgut microbiota and immunity, thereby promoting fungal infection. Sci. Rep..

[13] Polenogova O.V., Noskov Y.A., Yaroslavtseva O.N., Kryukova N.A., Alikina T., Klementeva T.N., Andrejeva J., Khodyrev V.P., Kabilov M.R., Kryukov V.Y. (2021). Influence of *Bacillus thuringiensis* and avermectins on gut physiology and microbiota in Colorado potato beetle: Impact of enterobacteria on susceptibility to insecticides. PLoS ONE.

[14] Dubovskiy I.M., Grizanova E.V., Whitten M.M.A., Mukherjee K., Greig C., Alikina T., Kabilov M., Vilcinskas A., Glupov V.V., Butt T.M. (2016). Immuno-physiological adaptations confer wax moth *Galleria mellonella* resistance to *Bacillus thuringiensis*. Virulence.

[15] Regev A., Keller M., Strizhov N., Sneh B., Prudovsky E., Chet I., Ginzberg I., Koncz-Kalman Z., Koncz C., Schell J. (1996). Synergistic activity of a *Bacillus thuringiensis* delta-endotoxin and a bacterial endochitinase against *Spodoptera littoralis* larvae. Appl. Environ. Microbiol..

[16] Liu Y.J., Shen Z., Yu J., Li Z., Liu X., Xu H. (2020). Comparison of gut bacterial communities and their associations with host diets in four fruit borers. Pest Manag. Sci..

[17] Gong Q., Cao L.J., Sun L.N., Chen J.C., Gong Y.J., Pu D.Q., Huang Q., Hoffmann A.A., Wei S.J. (2020). Similar gut bacterial microbiota in two fruit-feeding moth pests collected from different host species and locations. Insects.

[18] Zhang Y., Zhao T., Deng J., Zhou X., Wu Z., Su Q., Zhang L., Long Y., Yang Y. (2019). Positive effects of the tea catechin (-)- epigallocatechin-3-gallate on gut bacteria and fitness of *Ectropis obliqua Prout* (Lepidoptera: Geometridae). Sci. Rep..

[19] Caccia S., Di Lelio I., La Storia A., Marinelli A., Varricchio P., Franzetti E., Banyuls N., Tettamanti G., Casartelli M., Giordana B. (2016). Midgut microbiota and host immunocompetence underlie *Bacillus thuringiensis* killing mechanism. Proc. Natl. Acad. Sci. USA.

[20] Xia X., Sun B., Gurr G.M., Vasseur L., Xue M., You M. (2018). Gut microbiota mediate insecticide resistance in the diamondback moth, *Plutella xylostella* (L.). Front. Microbiol..

[21] Allegra E., Titball R.W., Carter J., Champion O.L. (2018). *Galleria mellonella* larvae allow the discrimination of toxic and non-toxic chemicals. Chemosphere.

[22] Ignasiak K., Maxwell A. (2017). *Galleria mellonella* (greater wax moth) larvae as a model for antibiotic susceptibility testing and acute toxicity trials. BMC Res. Notes.

[23] Sugeçti S., Tunçsoy B., Büyükgüzel E., Özalp P., Büyükgüzel K. (2021). Ecotoxicological effects of dietary titanium dioxide nanoparticles on metabolic and biochemical parameters of model organism *Galleria mellonella* (Lepidoptera: Pyralidae). J. Environ. Sci. Health C.

[24] Duman E.E., Gwokyalya R., Altuntas H., Kutrup B. (2022). Screening the immunotoxicity of different food preservative agents on the model organism *Galleria mellonella* L. (Lepidoptera: Pyralidae) larvae. Drug Chem. Toxicol..

[25] Coates C.J., Lim J., Harman K., Rowley A.F., Griffiths D.J., Emery H., Layton W. (2019). The insect, *Galleria mellonella*, is a compatible model for evaluating the toxicology of okadaic acid. Cell Biol. Toxicol..

[26] Cools F., Torfs E., Aizawa J., Vanhoutte B., Maes L., Caljon G., Delputte P., Cappoen D., Cos P. (2019). Optimization and characterization of a *Galleria mellonella* larval infection model for virulence studies and the evaluation of therapeutics against *Streptococcus pneumonia*. Front. Microbiol..

[27] Champion O.L., Wagley S., Titball R.W. (2016). *Galleria mellonella* as a model host for microbiological and toxin research. Virulence.

[28] Stephens J.M. (1962). A strain of *Streptococcus faecalis* Andrewes and Horder producing mortality in larvae of *Galleria mellonella* (Linnaeus). J. Insect Pathol..

[29] Johnston P.R., Rolff J. (2015). Host and Symbiont jointly control gut microbiota during complete metamorphosis. PloS Pathog..

[30] Krams I., Kecko S., Inashkina I., Trakimas G., Krams R., Elferts D., Elferts D., Vrublevska J., Jõers P., Rantala M.J. (2017). Food quality affects the expression of antimicrobial peptide genes upon simulated parasite attack in the larvae of greater wax moth. Entomol. Exp. Appl..

[31] Allonsius C.N., Van Beeck W., De Boeck I., Wittouck S., Lebeer S. (2019). The microbiome of the invertebrate model host *Galleria mellonella* is dominated by *Enterococcus*. Anim. Microbiome.

[32] Gooch H.C., Kiu R., Rudder S., Baker D.J., Hall L.J., Maxwell A. (2021). *Enterococcus innesii* sp. nov., isolated from the wax moth *Galleria mellonella*. Int. J. Syst. Evol. Microbiol..

[33] Hammer T.J., Moran N.A. (2019). Links between metamorphosis and symbiosis in holometabolous insects. Philos. Trans. R. Soc. B.

[34] Indiragandhi P., Anandham R., Madhaiyan M., Sa T.M. (2008). Characterization of plant growth-promoting traits of bacteria isolated from larval guts of diamondback moth *Plutella xylostella* (Lepidoptera: Plutellidae). Curr. Microbiol..

[35] Buchon N., Broderick N.A., Chakrabarti S., Lemaitre B. (2009). Invasive and indigenous microbiota impact intestinal stem cell activity through multiple pathways in *Drosophila*. Genes Dev..

[36] Shao Y., Chen B., Sun C., Ishida K., Hertweck C., Boland W. (2017). Symbiont-derived antimicrobials contribute to the control of the lepidopteran gut microbiota. Cell Chem. Biol..

[37] Broderic N.A., Raffa K.F., Handelsman J. (2006). Midgut bacteria required for *Bacillus thuringiensis* insecticidal activity. Proc. Natl. Acad. Sci. USA.

[38] Mason K.L., Stepien T.A., Blum J.E., Holt J.F., Labbe N.H., Rush J.S., Raffa K.F., Handelsman J. (2011). From commensal to pathogen: Translocation of *Enterococcus faecalis* from the midgut to the hemocoel of *Manduca sexta*. mBio.

[39] Raymond B., Johnston P.R., Wright D.J., Ellis R.J., Crickmore N., Bonsall M.B. (2009). A mid-gut microbiota is not required for the pathogenicity of *Bacillus thuringiensis* to diamondback moth larvae. Environ. Microbiol..

[40] Kryukova N.A., Mozhaytseva K.A., Rotskaya U.N., Glupov V.V. (2020). *Galleria mellonella* larvae fat body disruption (Lepidoptera: Pyralidae) caused by the venom of *Habrobracon brevicornis* (Hymenoptera: Braconidae). Arch. Insect Biochem. Physiol..

[41] Fadrosh D.W., Ma B., Gajer P., Sengamalay N., Ott S., Brotman R.M., Ravel J. (2014). An improved dual-indexing approach for multiplexed 16S rRNA gene sequencing on the Illumina MiSeq platform. Microbiome.

[42] Melekhina E.N., Belykh E.S., Markarova M.Y., Taskaeva A.A., Rasova E.E., Baturina O.A., Kabilov M.R., Velegzhaninov I.O. (2021). Soil microbiota and microarthropod communities in oil contaminated sites in the European Subarctic. Sci. Rep..

[43] Edgar R.C. (2013). UPARSE: Highly accurate OTU sequences from microbial amplicon reads. Nat. Methods.

[44] Edgar R.C. (2018). Accuracy of taxonomy prediction for 16S rRNA and fungal ITS sequences. PeerJ.

[45] Wang Q., Garrity G.M., Tiedje J.M., Cole J.R. (2007). Naive Bayesian classifier for rapid assignment of rRNA sequences into the new bacterial taxonomy. Appl. Environ. Microbiol..

[46] Pedregosa F., Varoquaux G., Gramfort A., Michel V., Thirion B., Grisel O., Blondel M., Prettenhofer P., Weiss R., Dubourg V. (2011). Scikit-learn: Machine Learning in Python. JMLR.

[47] Virtanen P., Gommers R., Oliphant T.E., Haberland M., Reddy T., Cournapeau D., Burovski E., Peterson P., Weckesser W., Bright J. (2020). SciPy 1.0: Fundamental algorithms for scientific computing in Python. Nat. Methods.

[48] Chertkova E., Kabilov M.R., Yaroslavtseva O., Polenogova O., Kosman E., Sidorenko D., Alikina T., Noskov Y., Krivopalov A., Glupov V.V. (2023). Links between soil bacteriobiomes and fungistasis toward fungi infecting the Colorado potato beetle. Microorganisms.

[49] Elpidina E.N., Vinokurov K.S., Gromenko V.A., Rudenskaya Y.A., Dunaevsky Y.E., Zhuzhikov D.P. (2001). Compartmentalization of proteinases and amylases in *Nauphoeta cinerea* midgut. Arch. Insect Biochem. Physiol..

[50] Gatehouse J.A. (2002). Plant resistance towards insect herbivores: A dynamic interaction. New Phytol..

[51] Anson M.L. (1938). The estimation of pepsin, trypsin, papain, and cathepsin with hemoglobin. J. Gen. Physiol..

[52] Noskov Y.A., Polenogova O.V., Yaroslavtseva O.N., Belevich O.E., Yurchenko Y.A., Chertkova E.A., Kryukova N.A., Kryukov V.Y., Glupov V.V. (2019). Combined effect of the entomopathogenic fungus *Metarhizium robertsii* and avermectins on the survival and immune response of *Aedes aegypti* larvae. PeerJ.

[53] Habig W.H., Pabst M.J., Jakoby W.B. (1974). Glutathione S-transferases. The first enzymatic step in mercapturic acid formation. J. Biol. Chem..

[54] Nicell J.A., Wright H. (1997). A model of peroxidase activity with inhibition by hydrogen peroxide. Enzyme Microb. Technol..

[55] Wong G.W., McHugh T.M., Weber R., Goeddel D.V. (1991). Tumor necrosis factor alpha selectively sensitizes human immunodeficiency virus-infected cells to heat and radiation. Proc. Natl. Acad. Sci. USA.

[56] Bradford M.M. (1976). A rapid and sensitive method for the quantitation of microgram quantities of protein utilizing the principle of protein-dye binding. Anal. Biochem..

[57] Polenogova O.V., Noskov Y.A., Artemchenko A.A., Zhangissina S., Klementeva T.N., Yaroslavtseva O.N., Khodyrev V.P., Kruykova N.A., Glupov V.V. (2022). *Citrobacter freundii*, a natural associate of the Colorado potato beetle, increases larval susceptibility to *Bacillus thuringiensis*. Pest Manag. Sci..

[58] García-Solache M., Rice L.B. (2019). The *Enterococcus*: A model of adaptability to its environment. Clin. Microbiol. Rev..

[59] Grau T., Vilcinskas A., Joop G. (2017). Probiotic *Enterococcus mundtii* isolate protects the model insect *Tribolium castaneum* against *Bacillus thuringiensis*. Front. Microbiol..

[60] Huang S., Han C., Ma Z., Zhou J., Zhang J., Huang L. (2017). Identification and characterization of a pyridoxal 5’-phosphate phosphatase in the silkworm (*Bombyx mori*). Comp. Biochem. Physiol. B Biochem. Mol. Biol..

[61] Unban K., Klongklaew A., Kodchasee P., Pamueangmun P., Shetty K., Khanongnuch C. (2022). *Enterococci* as dominant xylose utilizing lactic acid bacteria in eri silkworm midgut and the potential use of *Enterococcus hirae* as probiotic for eri culture. Insects.

[62] Li G., Xia X., Zhao S., Shi M., Liu F., Zhu Y. (2020). The physiological and toxicological effects of antibiotics on an interspecies insect model. Chemosphere.

[63] Klemens J.J., Meech R.P., Hughes L.F., Somani S., Campbell K.C. (2003). Antioxidant enzyme levels inversely covary with hearing loss after amikacin treatment. J. Am. Acad. Audiol..

[64] Bas E., Van De Water T., Gupta C., Dinh J., Vu L., Martínez-Soriano F., Láinez J., Marco J. (2012). Efficacy of three drugs for protecting against gentamicin-induced hair cell and hearing losses. Br. J. Pharmacol..

[65] Büyükgüzel E., Kalender Y. (2008). *Galleria mellonella* (L.) survivorship, development and protein content in response to dietary antibiotics. J. Entomol. Sci..

[66] Keleş V., Büyükgüzel K., Büyükgüzel E. (2021). The effect of streptomycin on survival, development, and some biochemical aspects of *Drosophila melanogaster*. Turk. J. Zool..

[67] Hu B., Hu S., Huang H., Wei Q., Ren M., Huang S., Tian X., Su J. (2019). Insecticides induce the co-expression of glutathione S-transferases through ROS/CncC pathway in *Spodoptera exigua*. Pestic. Biochem. Physiol..

[68] Chu C.C., Spencer J.L., Curzi M.J., Zavala J.A., Seufferheld M.J. (2013). Gut bacteria facilitate adaptation to crop rotation in the western corn rootworm. PNAS USA.

[69] Yuksekdag Z., Ahlatci N.S., Hajikhani R., Darilmaz D.O., Beyatli Y. (2021). Safety and metabolic characteristics of 17 *Enterococcus faecium* isolates. Arch. Microbiol..

[70] Pilon A.M., Olivera M.G.A., Guedes R.N.C. (2006). Protein digestibility, protease activity and post-embrionic development of the velvetbean caterpillar (*Anticarsia gemmatalis*) exposed to the trypsin-inhibitor benzamidine. Pestic. Biochem. Physiol..

[71] Pilon A.M., Olivera M.G.A., Pilon F.M., Guedes R.N.C., Olivera J.A., Fazollo A. (2009). Adaptacao da lagarta da soja *Anticarsia gemmatalis* Hübner (Lepidoptera: Noctuidae) ao inhibitor de protease benzamidine. Rev. Ceres..

[72] Scott I.M., Thaler J.S., Scott J.G. (2010). Response of a generalist herbivore *Trichoplusia ni* to jasmonate-mediated induced defense in tomato. J. Chem. Ecol..

[73] Meriño-Cabrera Y., Zanucio J.C., da Silva R.S., Solis-Vargas M., Cordeiro G., Rainha F.R., Campos W.G., Picanço M.C., de Almeida Oliveira M.G. (2018). Biochemical response between insects and plants: An investigation of enzyme activity in the digestive system of *Leucoptera coffeella* (Lepidoptera: Lyonetiidae) and leaves of *Coffea arabica* (Rubiaceae) after herbivory. Ann. Appl. Biol..

[74] Harrison R.L., Bonning B.C. (2010). Proteases as insecticidal agents. Toxins.

[75] Chikate Y.R., Tamhane V.A., Joshi R.S., Gupta V.S., Giri A.P. (2013). Differential protease activity augments polyphagy in *Helicoverpa armigera*. Insect Mol. Biol..

[76] Sarate P.J., Tamhane V.A., Kotkar H.M., Ratnakaran N., Susan N., Gupta V.S., Giri A.P. (2012). Developmental and digestive flexibilities in the midgut of a polyphagous pest, the cotton bollworm, *Helicoverpa armigera*. J. Insect Sci..

[77] Klementeva T.N., Polenogova O.V., Glupov V.V. (2022). Effect of an antibiotic on gut microbiota and activity of digestive and antioxidant enzymes of *Galleria mellonella*. Eur. J. Entomol..

[78] Oppert B., Kramer K.J., Beeman R.W., Johnson D., McGaughey W.H. (1997). Proteinase-mediated insect resistance to *Bacillus thuringiensis* toxins. J. Biol. Chem..

[79] Lightwood D.J., Ellar D.J., Jarrett P. (2000). Role of proteolysis in determining potency of *Bacillus thuringiensis* Cry1Ac δ-endotoxin. Appl. Environ. Microbiol..

[80] Palmer K.L., Godfrey P., Griggs A., Kos V.N., Zucker J., Desjardins C., Cerqueira G., Gevers D., Walker S., Wortman J. (2012). Comparative genomics of enterococci: Variation in *Enterococcus faecalis*, clade structure in *E. faecium*, and defining characteristics of *E. gallinarum* and *E. casseliflavus*. mBio.

[81] Repizo G.D., Espariz M., Blancato V.S., Suárez C.A., Esteban L., Magni C. (2014). Genomic comparative analysis of the environmental *Enterococcus mundtii* against enterococcal representative species. BMC Genom..

[82] Sillanpaa J., Nallapareddy S.R., Prakash V.P., Qin X., Hook M., Weinstock G.M., Murray B.E. (2008). Identification and phenotypic characterization of a second collagen adhesin, *Scm*, and genome-based identification and analysis of 13 other predicted MSCRAMMs, including four distinct pilus loci, in *Enterococcus faecium*. Microbiology.

[83] Granger M., van Reenen C.A.A., Dicks L.M.T. (2008). Effect of gastro-intestinal conditions on the growth of *Enterococcus mundtii* ST4SA, and production of bacteriocin ST4SA recorded by real-time PCR. Int. J. Food Microbiol..

[84] Mazumdar T., The B.S., Murali A., Schmidth-Heck W., Schlenker Y., Vogel H., Boland W. (2021). Transcriptomics reveal the survival strategies of *Enterococcus mundtii* in the gut of *Spodoptera littoralis*. J. Chem. Ecol..

[85] Kong H.G., Son J.S., Chung J.H., Lee S., Kim J.S., Ryu C.M. (2023). Population dynamics of intestinal Enterococcus modulate *Galleria mellonella* metamorphosis. Microbiol. Spectr..

