# Supplementary material for: A Diet with Amikacin Changes the Bacteriobiome and the Physiological State of Galleria mellonella and Causes Its Resistance to Bacillus thuringiensis
Source: Insects. 2023 Nov 17;14(11):889. doi: 10.3390/insects14110889 (PMC10672437; doi:10.3390/insects14110889)
Supplement: Supplementary file 1 [file insects-14-00889-s001.zip › Supplementary Figures 1-5.pdf]

A diet with amikacin changes the bacteriobiome and the physiological state of *Galleria mellonella* and causes its resistance to *Bacillus thuringiensis*

Olga V. Polenogova[1*], Tatyana N. Klementeva[1], Marsel R. Kabilov[2], Tatyana Y. Alikina[2], Anton V. Krivopalov[1], Natalya A. Kruykova[1], Viktor V. Glupov[1]

[1]Laboratory of Insect Pathology, Institute of Systematics and Ecology of Animals, Siberian Branch of Russian Academy of Sciences, 630091, Novosibirsk, Russia; red.klen@yandex.ru (T.N.K.); krivopalov@gmail.com (A.V.K.); dragonfly6@yandex.ru (N.A.K.); skif61@list.ru (V.V.G.)

[2]Genomics Core Facility, Institute of Chemical Biology and Fundamental Medicine, Siberian Branch of Russian Academy of Sciences, 630090, Novosibirsk, Russia; kabilov@niboch.nsc.ru (M.R.K.); alikina@niboch.nsc.ru (T.Y.A.)

Correspondence: ovp0408@yandex.ru (O.V.P.)

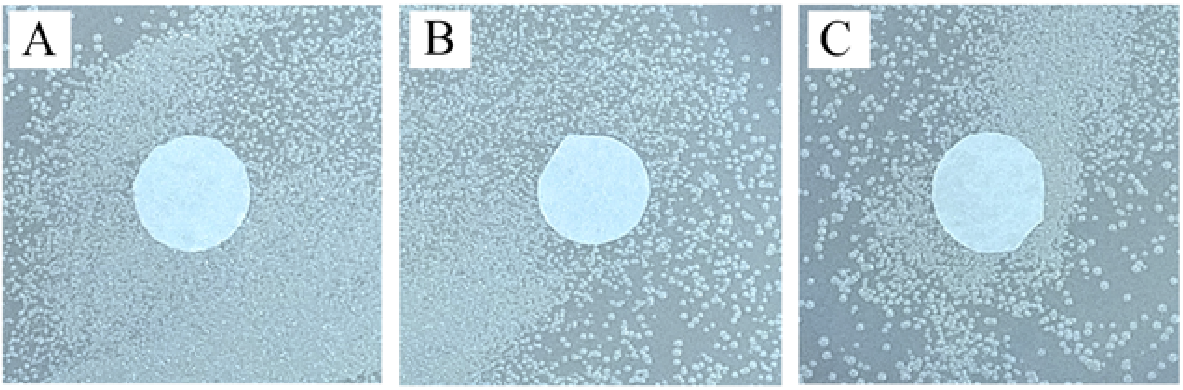

Figure S1. The sensitivity of *Enterococcus faecalis* GC1 [12] to amikacin (Synthesis, Russia) was determined *in vitro* using diffusion disks. The effect of amikacin on bacterial growth was assessed by plating 10 mm paper disks soaked in an antibiotic solution (A - 0.935, B - 1.87 and C - 3.75 mg/L) on to freshly plated bacterial culture lawns. The inhibition zones were measured after 48-hrs incubation at 28°C. The assays were performed in four replicates.

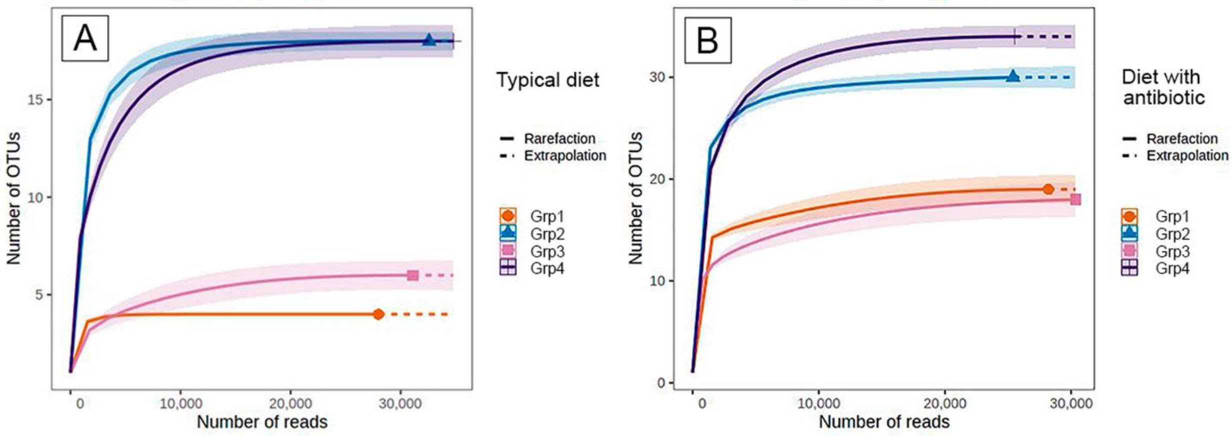

Figure S2. Rarefaction analysis of the investigated samples: typical diet (A) and diet with antibiotic (B).

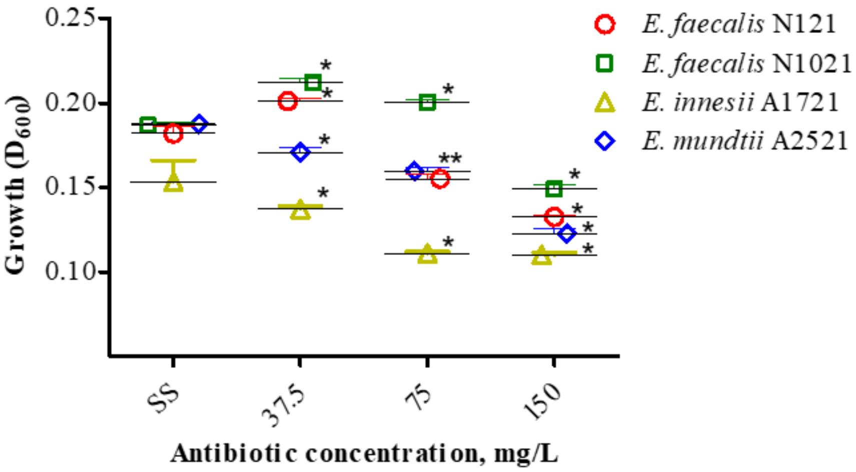

Figure S3. Growth of enterococci isolated of *G. mellonella* midgut bacteria (generation F18) after cultivation on typical artificial diet (*Enterococcus faecalis* N121 and *Enterococcus faecalis* N1021) and diet with antibiotic

(*Enretococcus innesii* A1721 and *Enterococcus mundtii* A2521). An overnight bacterial culture was incubated in nutrient broth in the presence of an antibiotic (37.5, 75, and 150 mg/L). Sodium chloride solution (SS) was used as a control. The optical density (D600) of the broth was evaluated after 24-hrs of cultivation at 28°C. Three replications were included for each variant of experiment. * – significant differences compared to SS (t-test, p= 0.03)

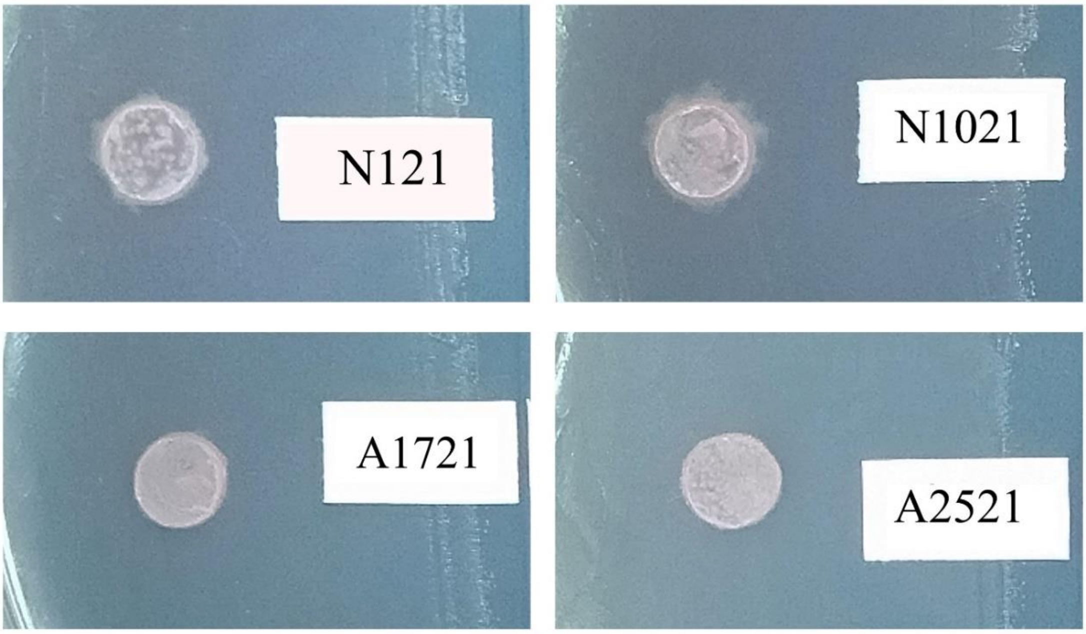

Figure S4. Interaction of enterococci isolated from the midgut *G. mellonella* (generation F18) and entomopathogenic bacteria *Bacillus thuringiensis* var. *galleria* 69-6 as assessed by the agar plug diffusion method on nutrient agar (pH 8.4). Petri dishes were incubated at 28°C. No bacterial growth inhibition was detected after 48-hrs of incubation at 28° C. Assays were performed in four replicates.

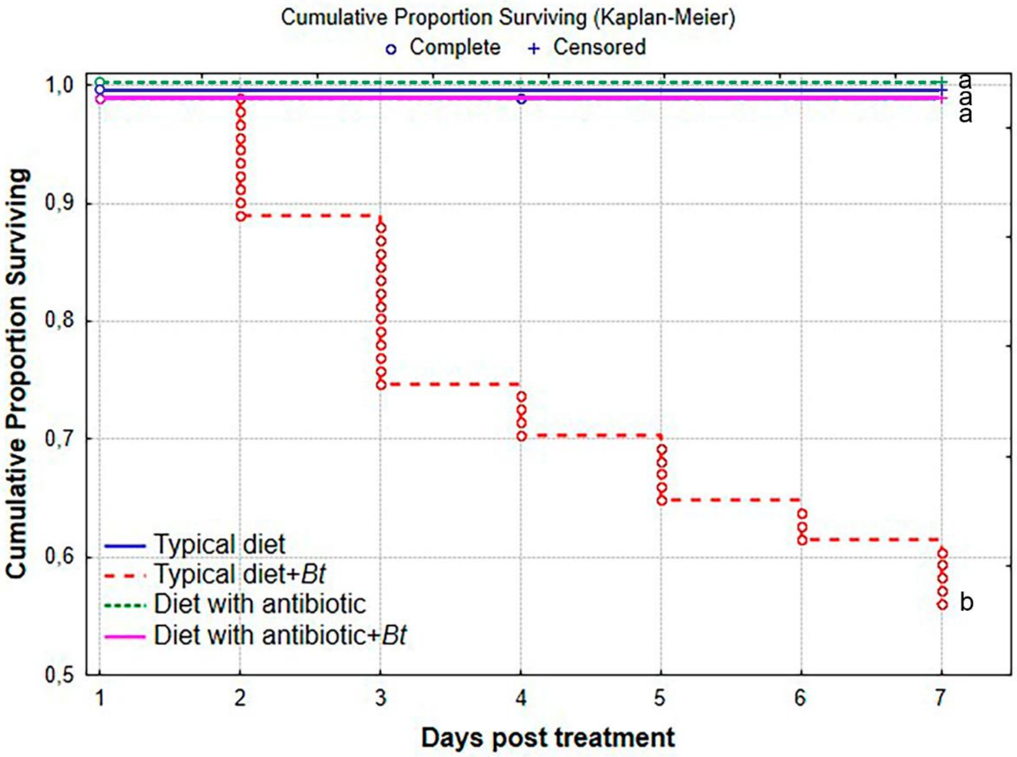

Figure S5. Survival of the F18 generation of *G. mellonella* after treatment with the entomopathogenic bacteria *Bacillus thuringiensis* var. *galleriae* 69-6 (2 × 10^8 spores and crystals/mL) *per os*. The F18 generation of *G. mellonella* larvae was reared on an artificial diet previously sterilized by gamma radiation (from newborn larvae to IV instar). Previously, during the seventeen generations, *G. mellonella* larvae were reared on the typical diet and the diet with antibiotic (final concentration of amikacin, 1.5 × 10^{-2}%). Before the experiment, the larvae were kept without food for 2 hrs, and an artificial diet with saline solution was prepared for the larvae in the control group. All the variants included three replicates (one replicate = 30 larvae). Different letters (a–b) indicate significant intergroup differences in wax moth survival ($p < 0.001$).
